# A Catalogue of Preparations Illustrative of Diseases of the Ear

**Published:** 1858-01

**Authors:** 


ART. IV.—*A Descriptive Catalogue of Preparations illustrative of the Diseases of the Ear.* In the Museum of JOSEPH TOYNBEE, F.R.S. London, 1857. pp. 127.

To Mr. Toynbee belongs the credit of being the first to investigate the diseases of the ear upon pathological principles. His labors were commenced in 1839, and have been zealously continued up to the present moment. The present brochure comprises a descriptive catalogue of 1659 dissections, involving an almost inconceivable amount of labor, to say nothing of the expense of the subjects and of the means of preserving so many preparations. Of these dissections 272 relate to the diseased ears of deaf persons the history of whose cases was known to Mr. Toynbee; 223 to the diseased ears of deaf persons of whose cases he had no knowledge; 654 to diseased ears to which no history was attached; and 510 to healthy ears. The following are the results which Mr. Toynbee has obtained from these researches:—

"I. The discovery of the existence of osseous tumors in the external meatus, and their structure.

"II. The detection of the presence of molluscous tumors in the external meatus; a disease which, in consequence of the accompanying discharge of mucus, has hitherto been confounded with 'otorrhœa.'

"III. The abolition of the terms 'otitis' and 'otorrhœa,' and the substitution of names indicating the tissue affected and the peculiar nature of the affection.

"IV. The discovery of the existence of the dermoid layer of the membrana tympani, which plays so important a part in the diseases of that membrane. It was previously supposed that the epidermoid layer was in direct contact with the fibrous layers.

"V. The ascertaining of the true relations of the two fibrous laminæ of the membrana tympani, and the existence and offices of the 'tubular tensor tympani ligament.'

"VI. The construction and application of the artificial membrana tympani in cases of perforation or destruction of the natural membrane.

"VII. The demonstration that the functions of the ossicles are analogous to those of the iris, modifying the access of sonorous vibrations as the latter does the undulations of light, attuning the labyrinth for the reception of either loud and harsh, or very low and delicate vibrations.

"VIII. The establishment of the existence, as a disease, of membranous and osseous ankylosis of the stapes to the fenestra ovalis, one of the most common causes of deafness.

"IX. The proof that the Eustachian tube remains always closed, except during the momentary act of swallowing, when its muscles cause it to open.

"X. The use of the 'otoscope' as a means by which the condition of the

Eustachian tube may always be diagnosed without the use of the Eustachian catheter.

"XI. The various diseases which give rise to caries of the petrous bone, and implicate in their progress the dura mater, the cerebrum, and the cerebellum, have been described, their nature and extent indicated, and means for their amelioration suggested."

Mr. Toynbee has published numerous essays and papers on the morbid anatomy and pathology of the ear, and he is now engaged in delivering an elaborate course of lectures upon the subject, which, when completed, will present the most valuable and scientific body of facts on aural medicine and surgery that has yet been given to the public. We shall hail the reprint of these lectures in this country with much pleasure.

